# *Aroplectrus dimerus* (Hymenoptera: Eulophidae), Ectoparasitoid of the Nettle Caterpillar, *Oxyplax pallivitta* (Lepidoptera: Limacodidae): Evaluation in the Hawaiian Islands

**DOI:** 10.3390/life14040509

**Published:** 2024-04-15

**Authors:** Juliana A. Yalemar, Walter T. Nagamine, Renato C. Bautista, Dexter Y. Cho, Larry M. Nakahara, Mohsen M. Ramadan

**Affiliations:** Hawaii Department of Agriculture, Division of Plant Industry, 1428 South King St., Honolulu, HI 96814, USA; yalemar@hawaii.edu (J.A.Y.); wnaloha@yahoo.com (W.T.N.); dexter.y.cho@hawaii.gov (D.Y.C.);

**Keywords:** host specificity, reproductive performance, colonization, parasitism rates, secondary parasitism, moth traps catch, Eulophidae, Limacodidae

## Abstract

The stinging nettle caterpillar, *Oxyplax* (syn. *Darna*) *pallivitta* (Lepidoptera: Limacodidae), is a serious invasive pest of agricultural products and a health hazard on the Hawaiian Islands first discovered in 2001. Nursery workers and homeowners have been stung by the caterpillars while handling their plants, especially rhapis palms (*Rhapis* sp.). Throughout its invaded range, it causes widespread damage, including the many cultivated and native palm species that have grown in Hawaii. Larvae contain urticating hairs that secrete a toxin, causing painful skin swelling and irritation on contact. Horticulture and nursery products impacted by the limacodid pest are estimated at $84.3 million (2018 value). Suppression efforts with pesticides and lure traps were ineffective, and the moth population continued to spread to major Hawaiian Islands (Hawaii, Kauai, Maui, Oahu). The introduction of specific biological control agents from the native region was thought to be the long-term solution for this invasive pest. Initial exploration in Indonesia and Thailand resulted in the introduction of a pupal ectoparasitoid, *Nesolynx* sp. (Hymenoptera: Eulophidae: Tetrastichinae), that was not specific. The oriental wasp, *Aroplectrus dimerus* Lin (Hymenoptera: Eulophidae: Eulophinae), idiobiont gregarious ectoparasitoid of the stinging nettle caterpillar, was introduced from Taiwan in 2004 for host specificity studies and biocontrol in Hawaii. Host range testing showed the parasitoid attacked only limacodid species, and it was approved for field release in 2010. The parasitoid identity, host specificity under containment facility conditions, reproductive performance, and colonization on the major infested sites were assessed. A total of 13,379 parasitoids were colonized on 162 release sites on four Hawaiian Islands. Evaluations were conducted using field surveys of larvae, pupal counts, and male lure traps. Field parasitism was thoroughly investigated on Oahu Island, averaging 18.9 ± 5.6% of 3923 collected larvae during 2010–2023. The numbers of male moths caught/trap/month were significantly reduced on Oahu Island (*p* < 0.05). Recently, the hyperparasitoid, *Pediobius imbreus* Walker (Hymenoptera: Eulophidae: Entedoninae), was detected, reducing the efficiency of *A. dimerus* in the field. The mean hyperparasitism of *A. dimerus* pupae was 27.3 ± 7.6% on Oahu Island. There was no detailed biological assessment for *A. dimerus* or its field evaluation available in scientific literature. Results were discussed regarding the potential use of *A. dimerus* in biocontrol elsewhere if the stinging nettle caterpillar was invaded in the future.

## 1. Introduction

The nettle caterpillar, *Oxyplax* (syn. *Darna*) *pallivitta* (Moore), is a new pest to the Hawaiian fauna that was first noticed in September 2001 after workers at a nursery on the island of Hawaii were being stung by a caterpillar while handling palms (*Rhapis* sp.), Arecaceae [1]. The moth was presumed to have entered the island on infested palm plantlets introduced from Taiwan [2]. Suppression efforts with pesticides were ineffective [3]. In January 2002, surveys pointed to its establishment on adjacent plantations where the caterpillars were feeding on *Cocos nucifera* L., *Chrysalidocarpus lutescens* Wendl, *Cordyline terminalis* Kunth, and *Dracaena* sp. Host plants were studied to include native plants that were also at risk [2,4]. Agricultural crops damaged by *O. pallivitta* include coffee, *Coffea arabica* L. (Rubiaceae), and macadamia, *Macadamia integrifolia* Maiden and Betche (Proteaceae) [3,5].

*Oxyplax pallivitta* became well-known in the Hilo area island of Hawaii (19°43′26.8032″ N and 155°5′12.5628″ W) and has gradually moved from the initial infestation site into Puna (19°32′30.4476″ N, and 155°6′3.6648″ W). It was found in Kona (19°38′23.9784″ N and 155°59′48.9588″ W) during September 2006 and at north Kohala (20°7′55″ N and155°47′38″ W) during February 2007, the new infestation sites likely resulting from movements of infested plants [1]. During June 2007, an influx at a plant sales outlet on the island of Oahu (21°27′59.99″ N and 157°58′59.99″ W) was discovered when laborers were being stung while holding *Dypsis lutescens* (Wendland) Beentje and Dransfield (Arecaceae). The source of this infestation was assumed to be the introduction of palms from a nursery on the island of Hawaii, where *O. pallivitta* was established. A similar situation appeared on Maui Island (20°47′54.1068″ N and 156°19′54.9264″ W) in July 2007, where a new infestation was found close to plant nurseries [6].

The feeding preference of many plants has increased its pest capacity in Hawaii since its introduction. Observations of feeding damage include both weedy (guinea grass, *Megathyrsus maximus* (Jacq.) B.K. Simon and S.W.L. Jacobs (Poaceae), mondo grass, *Ophiopogon japonicus* (Thunb.) Ker Gawl. (Asparagaceae), and ornamental plants grown in dwellings and agriculture lands [2,7]. Damage to garden plants and palm species grown in Hawaii could lead to business losses to the nursery trade and landholders. Horticulture and nursery products impacted by the limacodid pest are estimated at $84.3 million (National Agricultural Statistics Service 2018 value) [3]. Additionally, those possibly vulnerable to larval feeding are the endemic plant species [2,3]. The stinging spines of the larva, which cause itching, burning, welts, and blisters on contact with the skin, are a health hazard. Persons stung by *O. pallivitta* larvae usually increase in summer (May–October) due to moth population surges. Occurrences in residential communities result in landowners getting stung while doing work in their gardens. Symptoms vary, depending on a person’s sensitivity. In some cases, required admission to clinics [7,8].

The moth naturally occurs in Asia: China, Indonesia and Java, Japan, western Malaysia, Taiwan, Thailand, and Vietnam. Host plants in those areas include *Adenostemma* sp., *Areca* sp., *Breynia* sp., coconut, *Ficus* sp., grasses, maize, and oil palm [9]. In the native region, the moth is a minor insect pest of coconut palms, probably due to the existence of predators and parasitoids that are not present in Hawaii [10,11,12]. The currently accepted name of the moth in the Global Biodiversity Information Facility (GBIF) is *Oxyplax pallivitta* (Moore, 1877) https://www.gbif.org/species/11014495 (accessed on 7 April 2024) [13].

A biological control program was initiated by the Hawaii Department of Agriculture (HDOA) to survey the native Asian region for biocontrol agents and import them to Hawaii. Several parasitoids are recorded on Limacodid pests. The ectoparasitoid *Aroplectrus dimerus* was introduced in 2004 for evaluation. There were no described biological investigations in the scientific literature for *A. dimerus;* thus, host specificity and life cycle studies were conducted in the HDOA Insect Containment Facility (ICF). We report on the host specificity tests, investigate the biology and identity, colonization on the islands, field assessment on the parasitoid’s performance, and the effect of extant secondary parasitism. Male lure traps were evaluated to monitor population reduction over the years. Implications for biological control elsewhere were discussed.

## 2. Materials and Methods

### 2.1. Explorations and Origin of the Parasitoid Colony

Host plants infested by *O. pallivitta* species were located by one of us (LMN) during a survey in Taiwan at a Tien-wei nursery on 8 October 2004 (24°04′18.56″ N, 120°33′44.48″ E, 76 m AMSL). Parasitized larvae on ti plants (*Cordyline terminalis*), rhapis palms native to southern China and Taiwan, and mini coconut palms were collected. Adult parasitoids started emerging from some caterpillars. Additional live, unparasitized *O. pallivitta* larvae were collected at two Ping-tung nurseries and were used for the reproduction of the emerging parasitoids from Tien-wei. Parasitoids were placed in perforated plastic snap cap vials (9 drams, 33.4 mL), and honey and water were provided. A shipment of the parasitoids was hand-carried to Hawaii for host range study in the HDOA-ICF.

### 2.2. Identity of the Primary and Secondary Parasitoids

The two parasitoids associated with *O. pallivitta* in the field were examined. Detail diagnostic features are reported using keys and descriptions of Lin 1963 [14] for *Aroplectrus dimerus* and Khan and Shafee 1982 [15] for *Pediobius imbreus*. Parasitoids were collected on plants infested by *O. pallivitta* on Oahu Island. Initial identification of the primary parasitoid was made as *Aroplectrus dimerus* Lin (Hymenoptera: Eulophidae) by Dr. Chao-dang Zhu on 6 December 2004, a taxonomist of the oriental species of Chalcidoidea at the Institute of Zoology, Chinese Academy of Sciences, Beijing, Peoples Republic of China, who related the Taiwan specimens with those at the Natural History Museum, London and completed the identification.

The photographs of card-mounted specimens produced in this report are taken with a digital camera (Olympus Tough, TG–5) attached to a Leica M 125 stereomicroscope.

### 2.3. Host Rearing

*Oxyplax pallivitta* larvae were reared in cages (42 × 42 × 62 cm, 70 mesh) and provided with green leaves of *Cordyline fruticosa* (L.) A. Chev, (Asparagaceae); or iris, *Crocosmia × crocosmiiflora* (Lemoine) N.E.Br.; (Iridaceae). After pupating and adult emergence, five pairs were put in a one-gallon glass jar with leaves (3.8 L). The newly emerged pairs were held for mating and egg-laying in the jar provided with honey drops and water. A bunch of ti or iris leaves, inserted into a flower tube vial (1.5 × 7.0 × 1.5 cm), was placed into the jar. Most eggs were laid on the glass; the first instars crawled onto the ti leaves. Mature larvae were transferred to a screened cage (30 × 30 × 60 cm, 70 mesh) for continued feeding and development.

A larval disease, identified as a cytoplasmic polyhedrosis virus (CPV), later became established in the HDOA-ICF; this was eliminated by rearing fewer larvae per cage (20–30 larvae).

### 2.4. Parasitoid Rearing

*Aroplectrus dimerus* was raised in a one-gallon glass jar (3.8 L) containing 15 mature host larvae (L6–L10 instars) and five newly emerged female parasitoids. The spread of honey was a food source for the parasitoids (SUE BEE^®^ SPUN^®^ siouxhoney.com/sue-bee-spun-honey). Host larvae were exposed for seven days; then, the female wasps were removed to avoid extreme parasitism. A new generation of adult parasitoids commenced emergence two weeks after first exposure.

### 2.5. Reproductive Parameters and Immature Measurements

The general longevity, fecundity, offspring sex ratio, and life cycle for *A. dimerus* were determined under insectary conditions (mean ± SEM, temperature 21.8 ± 0.12 °C, mean RH 70.2 ± 2.4%, photoperiod 12:12, D:L). Tests were performed on 40 newly emerged pairs of generations (July 2021–April 2022). Pairs were held in Petri dishes (150.0 Ø × 15.0 mm height) provided with honey, a water wick, and a daily larval host until female death. Reproductive parameters were determined by observations of exposed larvae individually isolated in Petri dishes for microscopic observation. Data on daily fecundity (number of eggs laid/female/day) until the death of female, adult emergence rates, offspring sex ratio, and longevity of males and females were assembled. Immature developmental periods and measurements for eggs, larvae, and pupae were calculated for 15 individuals using a Leica M 125 stereomicroscope provided with an eyepiece Micrometer.

### 2.6. Host Specificity Assessment

In Hawaii, there are no species in the family Limacodidae except *O. pallivitta*, and there are no species represented in its superfamily Zygaenoidea [16]. Consequently, there were no species closely related taxonomically for host testing. Twenty-five species representing 13 Lepidoptera families were checked to determine if the parasitoid *A. dimerus* would attack any non-target species (Table 1).

All host specificity testing for *A. dimerus* was conducted in the HDOA-ICF (minimum temperature 18.7 °C, maximum temperature 24.1 °C, minimum RH 61.6%, maximum RH 84.3%, photoperiod 12:12, D:L).

Host specificity assessments were based on no-choice tests. Ten larvae of a Lepidoptera test species were placed in a one-gallon glass jar (3.8 L) with their food source and exposed to five newly emerged *A. dimerus* females for a 24-h period. Larval food sources were available, placed in jars, and changed as needed. The control replicate had 10 *O. pallivitta* larvae and a bouquet of iris leaves as a food source. Honey and water were available for the wasps ad libitum.

After the exposure period, the number of parasitoid eggs was counted on tested larvae. The 10 test larvae with their respective food source were held until moth or parasitoid emergence occurred. The same procedure was followed with the control larvae; however, because of their long life cycle (≈10 weeks), [7] the larvae were only held for parasitoid emergence (≈2–3 weeks). Parasitoids’ ages were the same for a test and control replicate. Two replicates of 10 larvae each were completed for each Lepidoptera species, for a sum of 20 larvae investigated per species.

### 2.7. Colonization and Establishment Records on the Islands

**Figure 1:**
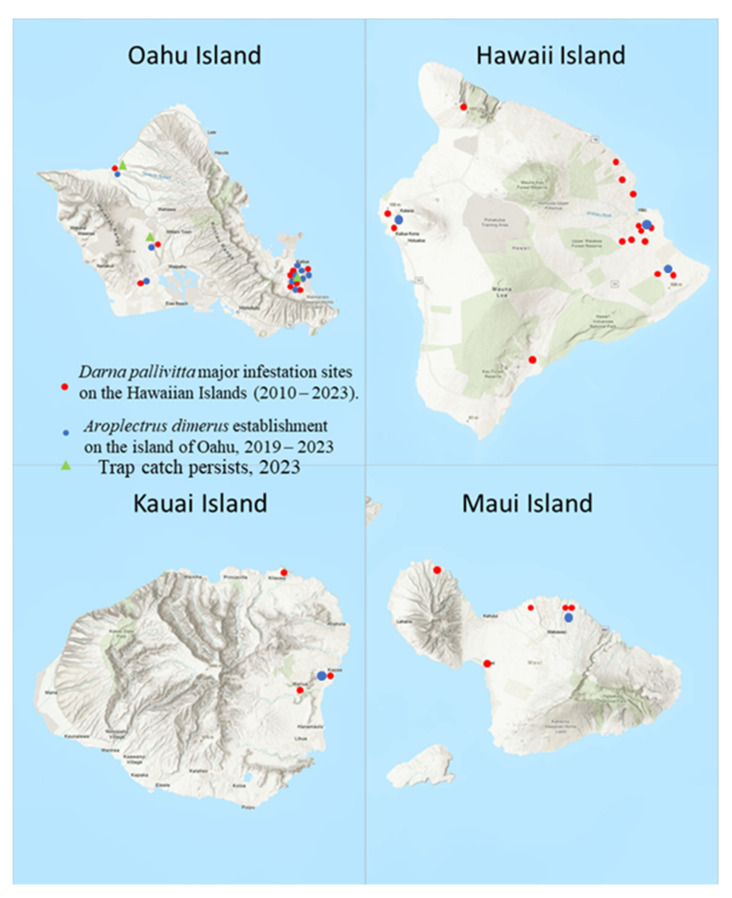
Map of major infestation sites, parasitoid colonization, and establishment on the Hawaiian Islands (sizes of the islands are not in scale). Sampling locations with GPS coordinates are shown in Tables. (Oahu Island GPS coordinates of 21°18′56.1708″ N, 157°51′29.1348″ W; Hawaii Island with the GPS coordinates of 19°44′30.3180″ N, 155°50′39.9732″ W; Kauai Island with GPS coordinates of 22°6′30.7548″ N, 159°29′48.3540″ W.; Maui Island with GPS coordinates of 20°47′54.1068″ N and 156°19′54.9264″ W. [https://www.latlong.net (accessed on 13 December 2023)].

Parasitoids were released periodically on major infested sites on four Hawaiian Islands (Hawaii, Kauai, Maui, and Oahu). Parasitoid releases were conducted during 2010–2023 as needed to obtain the parasitoid established. Release sets, range of wasps per lot, and total wasps released were recorded for every location by island. Release sites of major infested nurseries and homes were plotted on a map for all the infested islands. Geographic Coordinates (GPS) were recorded using Google Earth Pro online, version 7.3 (Figure 1).

### 2.8. Infestation Rates on Major Infested Sites on Oahu Island

A survey for the pupal count was conducted by eight HDOA staff members in June 2007 before the introduction of parasitoids. Collection of *O. pallivitta* cocoons at the Tkenaka Nursery, Kipapa Gulch, Central Oahu Island (21°27′32.80″ N, 158°00′57.32″ W, 215 m AMSL), from a group of 1000 infested potted Areca palm plants, *Dypsis lutescens*. Pupae were examined and counted. Empty cocoons with circular openings in the cocoons were considered as early moths’ emergence, and the rest of the cocoons were held in insectary cages (30 × 30 × 60 cm, 70 mesh) for possible pupal parasitoid emergence. Larvae from infested areas were collected periodically and held in Petri dishes (150.0 Ø ×15.0 mm height) with food provided until maturation to adult moths or parasitoid emergence.

### 2.9. Rates of Parasitism on Oahu Island

This evaluation was only possible on Oahu Island, which had the most parasitoid releases. The number of larvae collected, number of samples, and total *Oxyplax* larvae collected were held in Petri dishes under insectary conditions. Rates of parasitism were determined by the number of *O. pallivitta* larvae produced parasitoids and the percentages of parasitism with the hyperparasitoid.

Hyperparasitism rates from field collections were determined by turning over parasitized caterpillars and checking for the amber-colored pupae of the primary parasitoid (*A. dimerus*) vs. the darkened pupae caused by the hyperparasitoid, *Pediobius imbreus*.

To confirm *P. imbreus* as a secondary parasitoid, we conducted a test in a Petri dish (150.0 Ø × 15.0 mm height) with two nettle caterpillars previously parasitized by the primary parasitoid *A. dimerus*. The *Aroplectrus* larvae were allowed to feed on the caterpillar host for four days, two days before their pupation (i.e., before expelling the meconium, the larval fecal waste discharged before pupation). Two female wasps of the hyperparasitoid *P. imbreus* were introduced into the Petri dish, which was provided with honey and water. The female hyperparasitoids appeared to use their ovipositor to probe through the caterpillar body to locate the *Aroplectrus* larvae beneath the *Oxyplax* caterpillar. The *Aroplectrus* larvae darkened about eight days after being hyperparasitized. *Aroplectrus* larvae that are not hyperparasitized are normally amber-colored in the pupal stage.

### 2.10. Trapping

Synthetic Pheromone Lures, E7,9-10:COOnBu was synthesized by Pacific Agriscience, Singapore [17]. Red rubber septa were loaded with two amounts of E7,9-10:COOnBu, 250 lg for trap lures [17]. Traps were placed in infested areas and replaced once a month with the new lure. Male *O. pallivitta* were monitored in Hawaii, Maui, and Oahu islands during the years before and after the parasitoid’s release. Year and number of traps monitored in Hawaii: 2009 (29 traps); Maui: 2007 (93 traps), 2009 (11 traps); and on Oahu: 2009 (27 traps), 2011 (12 traps), 2021 (67 traps), 2022 (135 traps), 2023 (106 traps). Traps were deployed in infested sites on host plants, and lures were changed monthly with new traps per site.

### 2.11. Statistical Analysis and Vouchers

Analysis using One Way ANOVA and *t*-Test analysis were performed for the sex ratio (% female offspring). Means trap catch per month were summarized before and after evaluation and analyzed using ANOVA for Oahu Island trap catches. The survivorship of females and males was recorded daily and analyzed using ANOVA. Mean survival time was also estimated for both sexes. Realized fecundity was estimated using the total number of progeny (±SEM) produced by each female wasp over her lifetime, and oviposition rates were estimated using the mean numbers of progeny (±SEM) produced per day by each female wasp. Statistical analyses and calculations were carried out with JMP Version 11 (SW) [18].

Voucher specimens of the primary and secondary parasitoids were deposited at the Hawaii Department of Agriculture and Bishop Museum, Honolulu, Hawaii. Vouchers of *Aroplectrus dimerus* were deposited in Zhu, C.D., Huang collection, Guangxi, China. Specimens of *A. dimerus* are deposited in the Natural History Museum (London, UK), the collections at the National Museum of Natural History (Washington, DC, USA), and the National Museum of Natural Science (Taichung, Taiwan). Vouchers of *Pediobius imbreus* are deposited at the HDOA insect collection and the Bishop Museum, Honolulu, HI, USA.

## 3. Results

### 3.1. Exploration

The first attempt to explore *O. pallivitta* natural enemies was a partnership with Sam Ratulangi University located in Manado, Faculty of Agriculture, the Coconut Research Center in Manado, North Sulawesi, Indonesia, collected during September 2003 (1°27′19.94″ N, 124°49′37.62″ E, 28 m). Three limacodid species were collected for parasitism (*Pectinarosa alastor* Tams, 30 cocoons; *Thosea monoloncha* Meyrick, 27 cocoons; both from coconut hybrid leaves; and *Darna catenatus* Snellen, 352 cocoons from oil palm, *Elaeis guineensis* Jacq. yielded the parasitoid, *Nesolynx* sp. (Hymenoptera: Eulophidae), attacking the pupae as an ectoparasitoid emerging from the cocoon stage of *D. catenatus*. Testing in the HDOA-ICF presented this parasitoid to be a generalist as it parasitized two species of fly puparia (*Bactrocera dorsalis* (Hendel), (Diptera: Tephritidae); and *Trichopoda pilipes* (Fabricius) (Diptera: Tachinidae), under laboratory conditions. Therefore, additional host range testing was abandoned. An earlier shipment from Manado, North Sulawesi, by the same collector in September 2003 was not successful in producing any parasitoids; only dead larvae of limacodid arrived.

The second effort to gather potential biocontrol agents was made in collaboration with the National Biological Control Research Center in Thailand-Bangkok in June 2004. Collections showed *Parasa lepida* Cramer (Limacodidae) being attacked by an unidentified wasp (Hymenoptera: Braconidae). Nevertheless, the wasps died before any shipment made to Hawaii.

A third survey was conducted in Taiwan in October 2004 by one of us (LMN) in collaboration with the Agricultural Research Institute (TARI) and Ping-tung University.

*Oxyplax pallivitta* species was detected at a Tien-wei nursery on 8 October 2004 (Chang Hua province, 24°04′18.06″ N, 120°33′44.81″ E, 77 m AMSL). Parasitized larvae were found on ti plants, rhapis palms, and mini coconut palms. Wasps started emerging from the parasitized larvae. Collections of unparasitized *O. pallivitta* larvae were also made at two Ping-tung nurseries (22°48′40.69″ N, 120°35′46.00″75 m AMSL) and were used for reproduction of the parasitoid. Parasitoids were hand carried to Hawaii on 19 October 2004, for host range study in the HDOA-ICF. Wasps identified as *A. dimerus* reached HDOA alive and yielded 53 wasps (60.0% ♀♀) that were used for the colony rearing.

### 3.2. Identity of Parasitoids

*Aroplectrus* is a genus of Hymenoptera: Eulophidae: subfamily Eulophinae, tribe Euplectrini, recognized with four segmented funicles, normal size wings, and metatibia with one spur distinctly longer than basitarsus. Six species are recorded: *Aroplectrus areolatus* (Ferrière), *A. contheylae* Narendran, *A. dimerus* Lin, *A. flavescens* (Crawford), *A. haplomerus* Lin, and *A. noyesi* Narendran [19,20].

**Figure 2:**
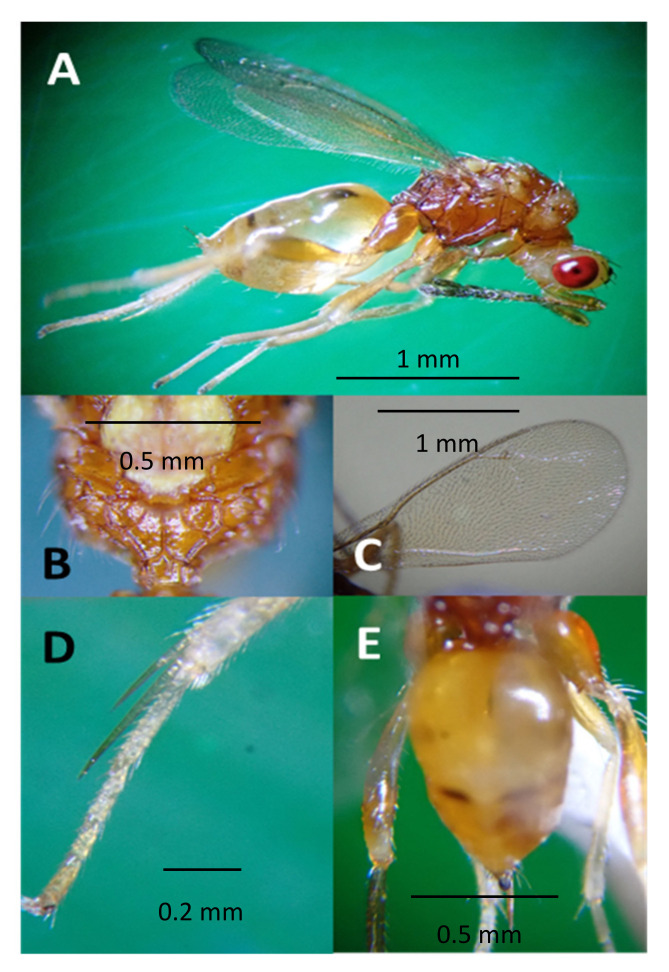
*Aroplectrus dimerus* (**A**) female side habitus, curved down mesosoma in profile along the dorsal margin, overall body color yellow and reddish, scape longer than eye, head narrower than mesosoma, smooth hind coxae; (**B**) scutellum finely granulate with longitudinal carinae, propodeum, median carina weak, submedian areola divided completely into two sectors by a continuous oblique carina; (**C**) forewing hyaline, densely pilose veins brownish wing post-marginal vein longer than stigmal vein; (**D**) elongate metatibial spur longer than basitarsus, not reaching apex of second tarsal segment; (**E**) gaster, female metasoma shorter and narrower than mesosoma, oblong-ovate in dorsal view unicolor, gaster showing dark bands and black ovipositor sheath, ovipositor exerting beyond abdominal apex, smooth hind coxae (pictures taken using MMR).

**Figure 3:**
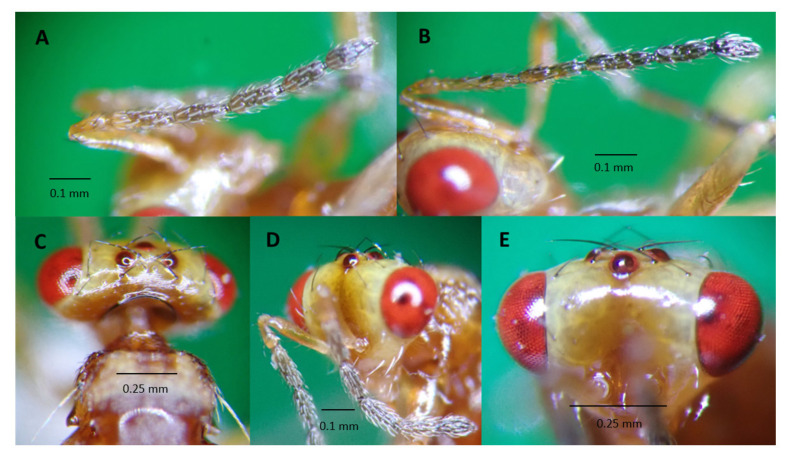
*Aroplectrus dimerus* (**A**) female antenna, funicle 4 segmented and a clava, F1 4X longer than broad, antenna with reddish scape, darker on funicle, female antenna, clava as long as F4; (**B**) male antenna showing slender funicle and shorter clava, antennae more slender, club broader than funicle 1; (**C**) showing vertex and yellow pronotum, head dorso-posterior view showing occipital carina feature, and quadrate pronotum with two side-long sitae in the middle; (**D**) dorso frontal view of head showing scape longer than eye and facial epistomal suture distinct straight, vertex with few black sitae and sparse cilia, malar space smooth shorter than eye, antenna with scape much longer than eye; (**E**) head frontal facial view showing head wider than head length (pictures taken using MMR).

*Aroplectrus dimerus* has a general body color yellow, scape distinctly longer than eye, recognized with head much narrower than thorax (Figure 2 and Figure 3), head in front view wider than high, clava shorter than FI (Figure 3A), submedian propodeal areola divided completely into two sectors by a continuous oblique carina (Figure 2B), hind basitarsus much longer than second tarsal segment, hind tibial spurs very long and strong reaching apex of second tarsal segment (Figure 2D), scutellum without lateral grooves, and propodeum with a single strong median carina [14,19,20].

Recorded hosts are *Parasa bicolor* (Lepidoptera: Limacodidae) and Cochliidae. This Oriental species is reported to occur in mainland China: Guangxi (Napo, Pingxiang), Hainan (Yaxian), India, Indonesia, Philippines, Taiwan, and Thailand [14]. The parasitoid is specific to members of the family Limacodidae (7 species in 3 genera) [20]. In Hawaii, there are no native limacodids.

*Pediobius* is a large genus of Hymenoptera: Eulophidae, with 217 known species worldwide [20,21]. It is composed of small wasps (0.8–1.6 mm), characterized by having propodeum medially with two subparallel carinae diverging posteriorly and with distinct plicae; frontofacial sutures distinct, petiole in most species with ventrally pointed extension [22,23,24].

**Figure 4:**
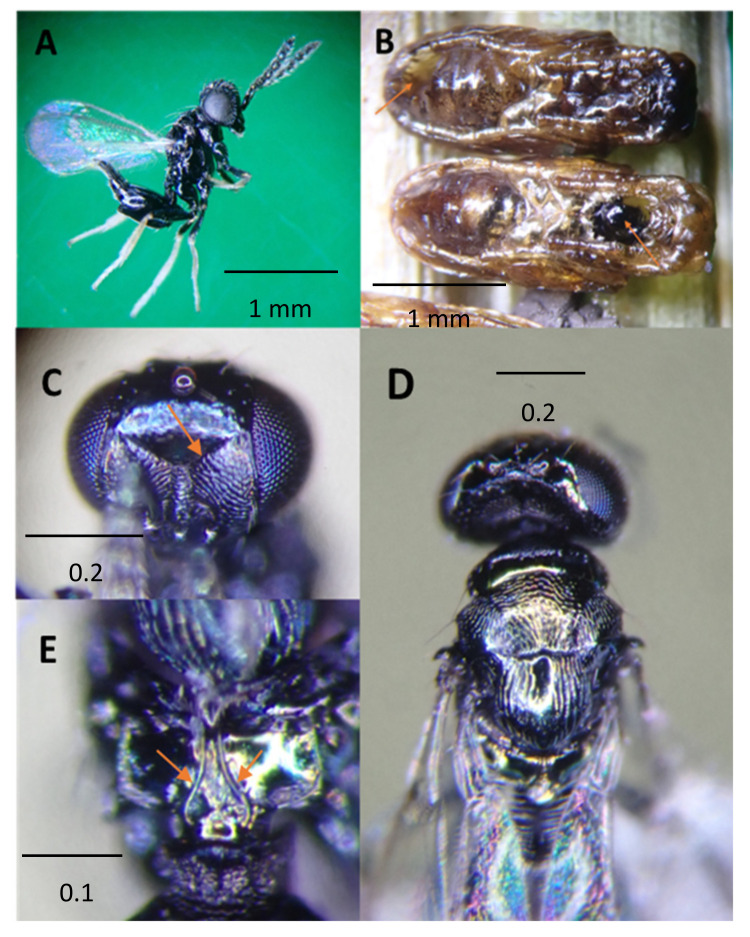
*Pediobius imbreus*, (**A**) side habitus of female, has a body mostly dark with less metallic reflections, antennae inserted at the lower level of eyes, coxae, trochanters, femora black, tibiae, and tarsus coloration varied between specimens in HDOA collection, some specimens with all dark or all white, with or without metallic bluish reflections; (**B**) exit holes from pupae of *Aroplectrus dimerus* (red arrows on exit holes anterior with hyper pupal molt, and posterior of pupa); (**C**) head front view showing transverse frontal suture extended close to compound eyes; (**D**) scutum reticulate, scutellum with longitudinal reticulate sculpture having a median narrow, smooth band, broad head pronotum, and reticulate sculptured mesothorax; (**E**) propodium with divergent middle carina and lateral propodeal plicae. Propodeum short, with submedian carinae diverging posteriorly (pictures taken using MMR).

Adult *Pediobius imbreus* are hyperparasitoids collected from Waimanalo, Oahu Island, and are mostly dark with less metallic reflections; the female ≈ 1.6 mm in body length. The original description [24] and a redescription [25] of *P. imbreus*, indicated normal coloration with a blue-green iridescence that matches older specimens in the HDOA insect collections dated between 1917–1951. However, the latest specimens from Waimanalo, Oahu, have a yellow-green iridescence. This difference may be a color variant. Color variation in specimens collected in 2023, as depicted in Figure 4, differs in leg coloration. Similarly, the revised description by Kerrich 1973 acknowledged the variation in leg and body coloration [25].

The diagnosis of *P. imbreus* includes a V-shaped, generally complete frontal sulcus, its arms reaching inner eye margins, separated or somewhat fused, characterized by the relatively wide and robust head with an elongated and narrowed lower face. Antennae are attached near or below the lower eye margins (Figure 4A). Propodeum with two submedian carinae diverging posteriorly [26], Figure 4E.

*Pediobius imbreus* hyperparasitoid adults emerged about 3–5 days longer than cited in Indian literature [27], but the life cycle will probably vary for different hosts. Additionally, this test was performed in an insectary air-conditioned laboratory, so the cooler temperature may have slowed the wasp development and increased the life cycle duration. Unlike what we observed in Hawaii, this parasitoid was reported as a primary parasitoid of the Limacodid species in India [28].

### 3.3. Life History and Reproductive Performance

**Figure 5:**
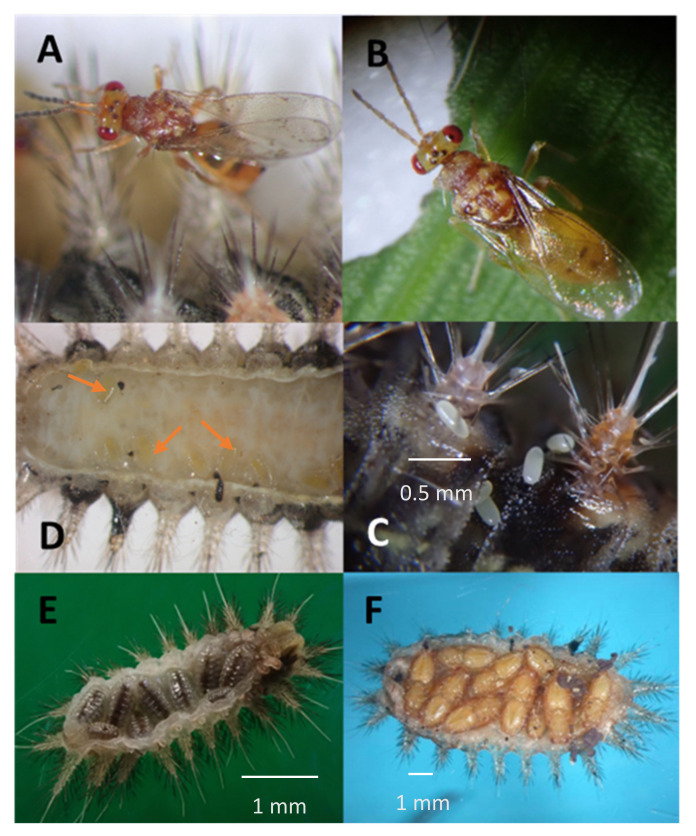
(**A**) female *Aroplectrus dimerus* on the host larva; (**B**) female dorsal view showing color peculiarity; (**C**) eggs laid on host larva between scolli; (**D**) first instars *A. dimerus* migrate to the underside of host larva (arrows point at first instars, black marks are female stinging marks to paralyze the host before oviposition not the feeding wounds by larvae); (**E**) mature larvae consume the host still with uncharged prepupal meconia; (**F**) pupae of the parasitoid underneath the host‘ cadaver, dark material between pupae are the vacated meconia. Photos credited to MMR and WTN.

*Aroplectrus dimerus* is a biparental, synovigenic species. It is an idiobiont, gregarious ectoparasitoid, typically 5–10 wasps developing from a single host larva, depending on the host instar (Figure 5E,F and Supplementary Materials). The female first stings the host larva, inserting its ovipositor at the edges of the ventral side. Melanized oviposition marks can be seen via microscopic examination on the belly side of the larva (Figure 5A). The *O. pallivitta* larva attacked by a wasp regurgitates a brown liquid. Sometimes, this killed the female parasitoid. The female wasp deposits individual eggs on the surface of the host larva, inserted between segments (Figure 5C). The parasitized larva becomes arrested within two days and stays stuck to the leaf substratum. The eggs hatch within two days under laboratory conditions (2.0 ± 0.0 days, *n* = 14). The first instar migrates to the belly of the host larva (Figure 5D) and feeds externally for 4.5 ± 0.14 days, *n* = 14. Larvae remain concealed under the host’s body (Figure 5E). One day prior to pupation, the parasitoid meconium is expelled as a dark brown substance (Figure 5F). The pupae developed in 5.6 ± 0.2 days, *n* = 14 (Figure 5F), and the adults afterward started to emerge. The total lifespan is 10.6 ± 0.2 days, *n* = 54 under laboratory conditions (Table 2).

**Figure 6:**
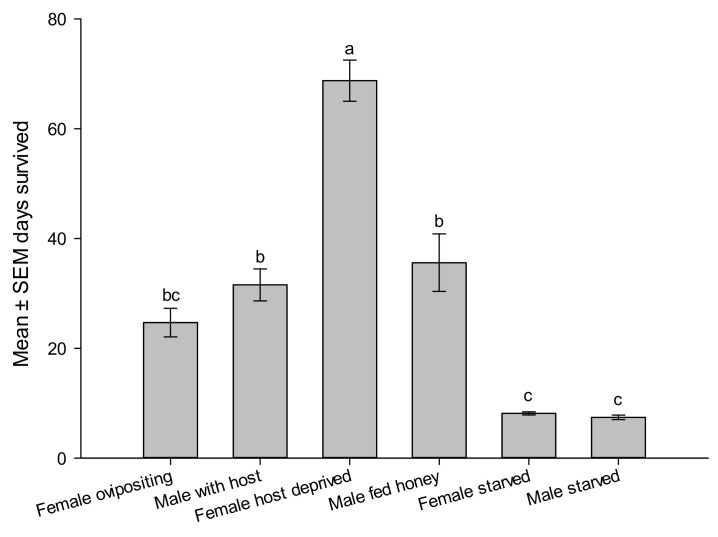
Survivorship of male and female *Aroplectrus dimerus* under laboratory conditions. All wasp categories fed honey and had access to water, except starved wasps. Different letters on top of bars indicate significant differences (ANOVA, *p* < 0.0001).

Reproductive parameters showed that females are readily mated as they emerge and ready for oviposition on the second day of emergence. The female continued to lay eggs for a week with a peak oviposition of 7.5 ± 0.8 per day and peak laid eggs of 9.5 ± 0.5 eggs/day, n = 40. Realized fecundity was 41.7 ± 4.1 eggs/female, n = 40, and 58.2 ± 3.0% emergence rate, n = 15. The longevity of ovipositing females was 24.7 ± 2.6 days shorter than male longevity of 31.5 ± 2.9 days. Males survived longer than ovipositing females (t_78_ = 1.750, *p* = 0.0420). However, host-deprived females survived significantly longer than males or any other category of wasp supplied by honey and water, sometimes reaching up to >2 months (Figure 6). Mated males survived the same periods as unmated honey-fed males. Starved wasps died after one week if not given honey or water sources. Feeding on honey significantly increased the survivorship of males and females (Figure 6).

Percentage sex ratio of female offspring (63.2 ± 3.0% ♀♀, n = 40) was significantly higher than % male offspring (t_46_ = −6.166, *p* < 0.0001), Table 2.

### 3.4. Host Specificity Tests

Choice test of host specificity demonstrated that females *A. dimerus* did not oviposit eggs on any investigated larvae of the non-target Lepidoptera species (Table 3). So, there was no wasp development. In all tests, larvae examined showed no evidence of oviposition marks due to probing.

Parasitism was documented in all the control (*O. pallivitta*) replicates. Analysis using One Way ANOVA showed a significant difference (*p* < 0.05) for parasitism among all non-target Lepidoptera species compared with their controls. The number of *O. pallivitta* larvae parasitized for a pair of replicates (n = 20 larvae) ranged from 40–85%, with an average of 4–7 wasps emerging per parasitized larva. The sex ratio of offspring showed that all females were mated (Table 3).

### 3.5. Colonization Records on the Islands

Parasitoids were released widely in Hawaii for several years, from 2010–2013. The establishment is now recorded throughout the Hawaiian Islands (Figure 1). By 2011, infestation reports showed that pest numbers had declined by 80–100% in HDOA survey sites (J. Yalemar, pers. comm., Hawaii Department of Agriculture). The total number of wasps released on four Islands was 13379 in 162 release sites in major infested areas from June 2010–December 2022 (Table 4, Figure 1).

### 3.6. Field Parasitism and Establishment

Total samples of larvae were collected on Oahu Island, where 3923 larvae had a mean of 18.9 ± 5.6% parasitism by *A. dimerus*. The culprit hyperparasitoid *P. imbreus* had a mean rate of 27.3 ± 7.6% parasitism (Table 5). Initial hyperparasitism on Oahu nursery during July–September 2010: revealed that out of 100 larvae collected had hypers in 46.8 ± 12.8% of collected larvae during four dates in July–September 2010 surveys at Waimanalo infested sites, n = 4. Hyperparasitism was also found on Kailua, Kona, Hawaii Island (19°43′05.05″ N, 155°59′49.65″ W, 438 m AMSL) on 20 September 2021. No record of hyperparasitism was reported from other islands.

### 3.7. Male Trap Catches and Pupal Infestation

**Figure 7:**
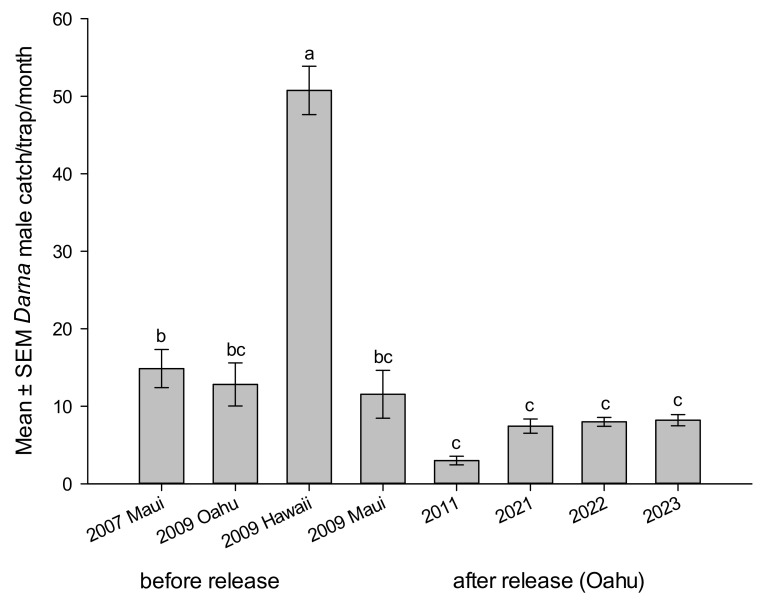
Mean number of *Oxyplax pallivitta* lured into male pheromone traps per month on the Hawaiian Islands before parasitoid release during 2007, 2009, and after parasitoid establishment during 2011, 2021–2023 on Oahu Island. Different letters on top of bars indicate significant differences (ANOVA, *p* < 0.0001).

Pheromone-baited traps are routinely used to monitor *O. pallivitta* abundance and spread and to help document population changes associated with releases of its parasitoid, *A. dimerus* [29]. The number of males caught/trapped per month was recorded on major infested sites on the islands. The mean number of males caught per month during the years was reported before and after the parasitoid liberations. Results indicated a significant reduction in trap catches during the years before and after parasitoid releases on Oahu Island (2009 vs. 2011–2023) (F_1,478_ = 81.515, *p* < 0.0001), Figure 7.

The mean number of *O. pallivitta* lured into male pheromone traps per month on the Hawaiian Islands before parasitoid release during 2007, 2009, and after parasitoid establishment during 2011, 2021–2023 on Oahu Island was significantly different (F_7,472_ = 31.643, *p* < 0.0001), Figure 7. Trends in moth abundance of *O. pallivitta* on Oahu and neighboring Islands indicated a significant reduction in the pest. Trap catch after the release of parasitoid (n = 320, mean 7.76 ± 0.39 moths) vs. trap catch before the release (n = 160, mean 22.83 ± 2.23 moths), was significantly different (t ratio 6.663, df = 169.041, *p* < 0.0001).

**Figure 8:**
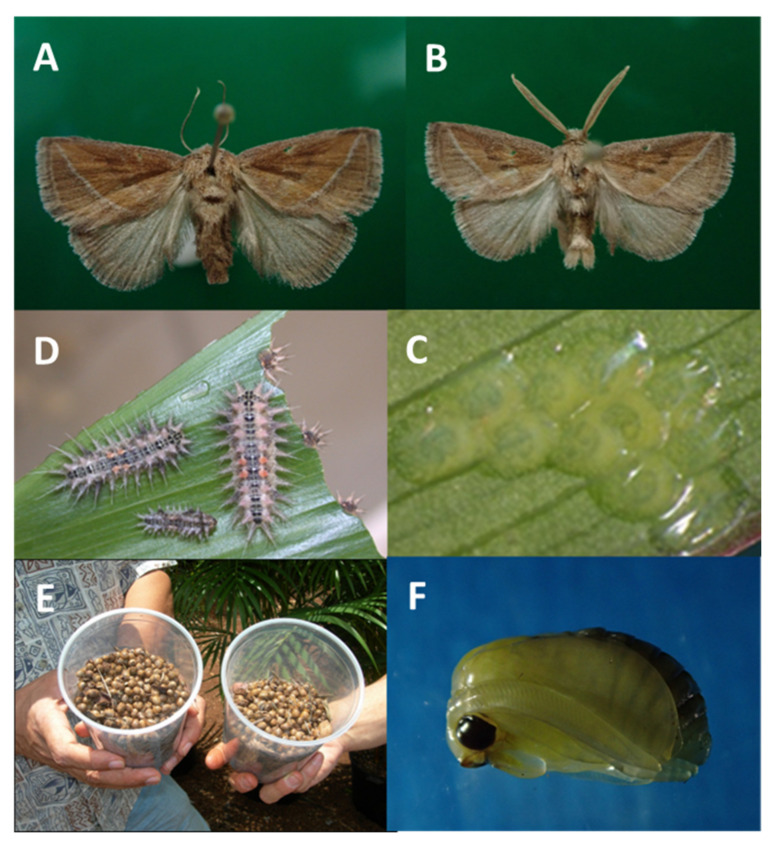
*Oxyplax pallivittus*: (**A**) female habitus; (**B**) male habitus see bipectinate antennae and end of abdomen; (**C**) flat eggs (1.6 mm length); (**D**) stinging larvae (L6–L10); (**E**) spherical cocoons collected from Oahu nursery in thousands in 2007 (6.5 mm Ø); (**F**) male pupa removed from the cocoon. Photos taken using MMR, WTN.

Hand removal of pupae was a rigorous task, and the result was a limited degree of control. Up to 50 cocoons have been removed from a single infested potted plant. The plants had been sprayed with Talstar (https://www.pedchem.com/products/talstar-pro-insecticide?variant=40105878847642, accessed on 2 January 2024). The survey in that nursery revealed a high infestation within the two-day survey, and some cocoons were empty (700 cocoons/two-day survey). The total number of plants examined was 1308 (327 ± 31.7 plants/day), had a total of 17,733 sound pupae (4433.3 ± 949.6 cocoon/day), and a mean of 13.56 ± 2.87 cocoons/plant. This survey was conducted before the introduction of parasitoids and illustrates the magnitude of infestation and how the parasitoid mitigated that influx of infested plants, Figure 8E.

## 4. Discussion

We report for the first time in literature on various aspects of host range testing, reproductive biology, eventual releases, and field parasitism of the *A. dimerus* in the Hawaiian Islands. Our rearing indicates a highly specialized parasitism in the laboratory and after many years of field releases. Besides *A. dimerus*, no other parasitoids were detected in Hawaii to mitigate the limacodid pest except for the egg parasitism by *Trichgramma*. However, the study did not continue to verify the effectiveness of egg parasitoids as biocontrol agents of Limacodidae. Parasitism of eggs by *Trichogramma papilionis* (Nagarkatti) was recorded in Hawaii by Conant et al. 2006 [30] before the introduction of *A. dimerus*. Mean parasitism calculated for 70 sentinels exposed egg batches was 4.4 ± 2.19% parasitism during September 2003–December 2004 in the Panaewa area, Hawaii Island [30]. The number of eggs per survey lot ranged from 1–162 eggs [2]. No additional *Trichogramma* have ever been discovered in March 2006. This could be another biotic factor undetermined in recent years.

Another unexplored mortality factor for this pest is the larval disease cytoplasmic polyhedrosis virus (CPV). Apparently, the disease was much established in the larval population of *O. pallivitta* under laboratory conditions. Out of 212 field-collected larvae, a mean of 26.6 ± 9.8%, n = 13, were diseased by CPV [2].

The reproductive parameters of this parasitoid could be essential information for developing a mass-rearing protocol. Field colonization and determination of rates of parasitism under field conditions has greatly enhanced our knowledge of this species under field conditions. No biological assessments were published in the information data for all the tribe Euplectrini [19,20].

*Aroplectrus dimerus* is known from China, India, Philippines, and Taiwan [10,30,31,32]. Host records are limited to larvae of Limacodidae [31,32,33,34]. None of the published records listed *Oxyplax pallivitta* as a host prior to this report during exploration and our host testing. *Oxyplax palllivitta* was discovered in Okinawa, Japan, and there has been no report of the utilization of parasitoids [35].

*Aroplectus dimerus* has adjusted well to parasitize *O. pallivitta* in Hawaii. Prior to oviposition, the female injects a venom that inhibits further ecdyses of the host, which hinders further molting of the caterpillar to advanced instars. The parasitoid larvae suck hemolymph out of the caterpillar, leaving a dried carcass of the larvae (Figure 5). Mature larvae of the parasitoid are protected by the dried host remains; therefore, they do not spin cocoons as other members of the tribe Euplectrini [23]. Euplectrini are polyphagous or oligophagous solitary or gregarious ectoparasitoids of lepidopterous larvae. The biology of most Euplectrini species has not been explored or considered for biocontrol elsewhere, with no data in the scientific literature from any of the native lands. *Aroplectrus* Lin (1963) has not been found in Sri Lanka closer to India [36].

There are six records of *Aroplectrus* on the Universal Chalcidoidea database (*Aroplectrus areolatus* (Ferriere) (from Indonesia, Sulawesi, and Malaysia ectoparasitoid on *Darna catenate*, and *Setora nitens*); *Aroplectrus contheylae* Narendran (from India, Kerala associated with Limacodidae on Arecaceae and *Cocos nucifera*); *Aroplectrus dimerus* Lin; *Aroplectrus flavescens* (Crawford) (from Philippines); *Aroplectrus haplomerus* Lin, (from Taiwan); and *Aroplectrus noyesi* Narendran, (from Thailand) [20]. Lin 1963 [14], in his description of *A. dimerus* did not record any hosts. His materials were collected by sweeping in open grassland and undergrowth of primary forests near Taipei, Taiwan. Leaving this report as the novel host association on *O. pallivitta* during our exploration in Taiwan.

The biology of this parasitoid and related species of *Aroplectrus* and the Euplectrini are not known from the literature [37]. *Aroplectrus dimerus* is distributed in India, Uttar Pradesh, the People’s Republic of China, Guangxi (Kwangsi), Philippines, and Taiwan in association with Limacodidae larvae. Among the host larvae are *Parasa bicolor*, *Penthocrates* sp., infesting mainly family Poaceae (*Saccharum officinarum*).

*Aroplectrus dimerus* has been recorded attacking six limacodid species in the Philippines [11,32]; these are *Darna mindanensis* Holloway, *Penthocrates albicapitata* Holloway, *P. rufa* Holloway, *P. rufofascia* Holloway, *P. styx* Holloway, and *P. zelaznyi* Holloway. The limacodid *Parasa bicolor* Walker is also a recorded host in India [30]. In this report, we included O. pallivitta as an important new host record for this parasitoid. Noyes (2019) listed no additional species as hosts in his database [20].

The culprit, *Pediobius imbrues*, is an adventive secondary parasitoid of *A. dimerus* larvae and pupae on Oahu and Hawaii Islands [16]. Most associated with species of Lepidoptera, Coleoptera, Diptera, and Hymenoptera as primary or secondary parasitoids [38]. Some species are known from spider egg sacs, where they may act as secondary parasitoids [38]. Limacodid host associations in North America are newly reported herein [39].

*Pediobius imbreus* (Hymenoptera: Eulophidae) was first recorded attacking the beneficial wasp *A. dimerus* shortly after its release on Oahu Island. Field collections of parasitized caterpillars during July 2010 showed the presence of the hyperparasitoid at a Waimanalo, Oahu nursery. Monthly collections of parasitized caterpillars from September–December 2010 yielded 50% hyperparasitism.

Biologically, species of *Pediobius* are quite diverse, acting as primary or secondary parasitoids, utilizing eggs, larvae, and pupae of species in the insect orders Coleoptera, Diptera, Hemiptera, Hymenoptera, Lepidoptera, Mantodea, and Thysanoptera [37,40]. *Pediobius imbreus* has also been recorded as a primary parasitoid of Limacodiid pests in India [27,28]. However, primary parasitism was not observed in the Hawaii infestations. *Pediobius imbreus*, with a natural incidence ranging from 2–10%, was observed in slug caterpillars affected gardens, and laboratory studies of this parasitoid revealed that it is a hyper parasitoid on *Bracon hebetor*, and its parasitization efficiency and longevity on slug caterpillar was recorded [41].

In a preliminary field efficacy study of *P. imbreus* against slug caterpillar *Macroplectra nararia* Moore, 41.4% parasitization was recorded. Therefore, the advantage of *P. imbreus* can be explored as a potential parasitoid for *M. nararia* in coconut plantations [41]. More observations are needed in Hawaii to determine if it has a primary mode of parasitism on *O. pallivitta*.

*Pediobius imbreus* was described from India, where it is recorded as a hyperparasitoid of Hymenoptera via their Lepidoptera hosts. For example, the coconut black-headed caterpillar, *Opisina arenosella* (Lepidoptera: Oecophoridae), is attacked by three species of primary parasitoids (*Apanteles taragamae* Viereck, *Bracon brevicornis* (Wesmael) (Braconidae) and *Goniozus nephantidis* (Muesebeck) (Bethylidae), but each of these were hyperparasitized by *P. imbreus* [27,28,41].

Indian literature showed the biology of *P. imbreus* in a laboratory study using *Bracon brevicornis* as a host. The female lays a single egg in the prepupal stage of the primary parasitoid. This generalist hyperparasitoid *P. imbreus*, a wasp that was already established in Hawaii and collected in 1917, reared from the cocoon of the braconid wasp *Bracon omiodivorus* Terry, a common parasitoid of caterpillars in Hawaii. Additionally, it was reared from the ichneumonid wasp *Cremastus* sp. (HDOA insect collection, 1949).

In the Hawaiian literature, *P. imbreus* was previously known as *Pleurotropis* sp. in 1917 and then later reported as *Pleurotropis detrimentosus*. According to Yoshimoto (1965), *P. imbreus* is established on all major islands of Hawaii [42]. It is unknown what impact *P. imbreus* could have on the effectiveness of *A. dimerus* in Hawaii. Further sampling of field-collected caterpillars will be necessary.

Lastly, we record the importance of our primary parasitoid, which we continue to release in Hawaii, beginning in 2010 as part of a biological control program against the invasive limacodid *Oxyplax pallivitta* [30,43,44]. Although neither *O. pallivitta* nor *A. dimerus* are currently known from the USA, it is likely that the limacodid will be introduced into California, given its history of interception at ports [45,46]. If this were to happen, it is possible that A. dimerus would be introduced to mitigate a new infestation [39].

Before this biocontrol agent was introduced to Hawaii, almost all the plants on the farm were heavily infested with pest larvae, so that foliage was marked with holes and many plants were close to defoliation. Six to eight months after the release of parasitoids, pest larvae were considerably suppressed, and moth trap catches had dropped dramatically.

Within a year or two of the release of *A. dimerus*, the pest had stopped spreading and was becoming difficult to find. Reported rates of parasitism were small and mostly affected by hyperparasitism. This makes us speculate if we need more new parasitoids for *O. pallivitta* in Hawaii. Perhaps more surveys for pupal and egg parasitism are required in Hawaii. At present, we are content because the public has no complaints [47]. Other islands are still free of infestation [48].

## 5. Conclusions

The collection of *O. pallivitta* on palm plants at a Taiwan nursery and *A. dimerus* during exploration in 2004 was a significant conclusion, adding a new host record for this prominent parasitoid unreported in the Chalcidoidea information data sets [20]. Natural enemies attained from the local range of a pest are more likely to have coevolved with its host and, therefore, have greater specificity.

*Oxyplax pallivitta* is still restricted to the islands of Hawaii, Maui, and Oahu [1,48]. Field collection from infested islands for redistribution of parasitoids for fast biocontrol releases could be achieved. This also could be rendered a quick recovery of established colonies for release in mainland USA if that need arose. *Oxyplax pallivitta* would probably be able to be established in southern California, Florida, Puerto Rico, the U.S. Virgin Islands, and southern Texas [49]. The larvae and cocoons were intercepted several times in California [49]. A survey in Hawaii in the sites mentioned in this manuscript would be effective in finding a new starter colony for possible release elsewhere.

Data on our rearing and reproductive performance on the *O. pallivitta* hosts are novel in literature. This wasp, however, has had only a limited effect on the nettle caterpillar population in Hawaii because of hyperparasitism, reaching up to 27%. It seems that *A. dimerus* is the only parasitoid recorded on this pest until the present time. No further study on the effect of many *Trichogramma* egg parasitoids on the islands was conducted.

## Figures and Tables

**Table 1:** Lepidopterous species used in host specificity assessments for the parasitoid *Aroplectrus dimerus*.

Family of Lepidoptera	Scientific and Common Name	Status and Source	Host Plant Common and Scientific Name
Erebidae	*Podomachla apicalis* (Walker) a leaf-feeder	Beneficial, Lab-reared	fireweed leaves, *Senecio madagascariensis*
Erebidae	*Secusio extensa* (Butler) a leaf-feeder	Beneficial Lab-reared	fireweed leaves, *Senecio madagascariensis*
Choreutidae	*Choreutis* sp. a leaf-tier	Pest Field-collected	weeping fig leaves, *Ficus benjamina*
Crambidae	*Diaphania nitidalis* Cramer pickleworm	Pest Lab-reared	cucumber flowers/fruit, *Cucumis sativa*, *Pipturis albidus*
Crambidae	*Omiodes blackburni* (Butler) coconut leaf roller	Endemic Lab-reared	coconut leaves, *Cocos nucifera*
Crambidae	*Udea stellata* (Butler) a leaf-feeder	Endemic Lab-reared,	mamaki leaves,
Ethmiidae	*Ethmia nigroapicella* (Sallmuller) kou leafworm	Pest Field-collected	kou leaves, *Cordia subcordata*
Geometridae	*Anacamptodes fragilaria* (Grossbeck), koa haole looper	Pest Field-collected	koa-haole leaves, *Leucaena leucocephala*
Geometridae	*Macaria abydata* Guenee koa haole moth	Pest Field-collected,	koa-haole leaves, *Leucaena leucocephala*
Lycaenidae	*Lampides boeticus* (Linnaeus) bean butterfly	Pest Field-collected	rattlepod beans, *Crotalaria* sp.
Noctuidae	*Achaea janata* (Linnaeus) croton caterpillar	Pest Field-collected	castor bean leaves, *Ricinus communis*
Noctuidae	*Agrotis* sp. a cutworm	Pest Lab-reared	cotton leaves, *Gossypium hirsutum*
Noctuidae	*Anomis flava* (Fabricius) hibiscus caterpillar	Pest Lab-reared	cotton leaves, *Gossypium hirsutum*
Noctuidae	*Heliothis virescens* (Fabricius) tobacco budworm	Pest Field-collected	love-in-a-mist flowers, *Passiflora foetida*
Noctuidae	*Pandesma anysa* Guenee a leaf-feeder	Pest Field-collected	opiuma leaves, *Pithecellobium dulce*
Noctuidae	*Spodoptera mauritia* (Boisduval), lawn armyworm	Pest Lab-reared	undetermined grass species
Nymphalidae	*Agraulis vanillae* (Linnaeus) passion vine butterfly	Pest Field-collected	passion vine leaves, *Passiflora edulis*
Nymphalidae	*Vanessa cardui* (Linnaeus) painted lady	Pest Field-collected	cheeseweed leaves, *Malva parviflora*
Pieridae	*Pieris rapae* (Linnaeus) imported cabbageworm	Pest Field-collected	broccoli leaves, *Brassica oleracea*
Plutellidae	*Plutella xylostella* (Linnaeus), diamondback moth	Pest Field-collected	broccoli leaves, *Brassica oleracea*
Pyralidae	*Hellula undalis* (Fabricius) imported cabbage webworm	Pest Field-collected	mustard cabbage leaves, *Brassica juncea*
Sphingidae	*Daphnis nerii* (Linnaeus) oleander hawk moth	Pest Field-collected	oleander leaves, *Nerium oleander*
Tortricidae	*Croesia zimmermani* Clarke a biocontrol agent	Beneficial Field-collected	blackberry leaves, *Rubus argutus*
Tortricidae	*Cryptophlebia ombrodelta* (Lower), litchi fruit moth	Pest Field-collected	undetermined legume species
Tortricidae	*Episimus utilis* Zimmerman a biocontrol agent	Beneficial Field-collected	x-mas berry leaves, *Schinus terebinthifolius*

**Table 2:** Reproductive attributes, developmental rates of mated *Aroplectrus dimerus* females, and measurements of immatures. The data are shown for 40 replicates in which a female was fed honey and allowed to oviposit on *Oxyplax pallivitta* larvae that were replaced every day.

Reproductive Parameter	*n*	Mean ± SEM	Range	Unit
Preoviposition period	40	1.60 ± 0.15	0–5	days
Oviposition period	40	7.77 ± 0.65	2–21	days
Post-oviposition period	36	10.86 ± 2.42	0–60	days
Age at peak oviposition	40	7.50 ± 0.79	2–24	days
Highest oviposition eggs/day	40	9.45 ± 0.55	4–19	number eggs
Egg deposition /day/larva	280	4.39 ± 0.26	0–19	number eggs
Fecundity total eggs deposited/female	40	41.67 ± 4.09	10–130	total eggs
Adult offspring emergence rate	15	58.20 ± 3.00	25–79	percentage
Female longevity	40	24.67 ± 2.61	8–76	days
Male longevity *	40	31.55 ± 2.93	8–90	days
Sex ratio (% females’ offspring) **	24	63.23 ± 3.00	8.9–85.7	% female
Sex ratio (% males’ offspring)	24	36.76 ± 3.03	14.2–91.04	% male
Life span	54	10.57 ± 0.19	9–13	days
Egg incubation period	14	2.0 ± 0.0	2	days
Larval developmental period	14	4.5 ± 0.14	4–5	days
Pupal developmental period	14	5.64 ± 0.20	5–7	days
Measurements of immatures				
Egg length	15	0.348 ± 0.005	0.32–0.40	mm
Egg width	15	0.139 ± 0.003	0.12–0.16	mm
Mature larval length	15	2.28 ± 0.137	1.25–3.0	mm
Mature larval width	15	0.88 ± 0.054	0.57–1.25	mm
Pupal length	15	2.78 ± 0.063	2.44–3.12	mm
Pupal width	15	0.95 ± 0.035	0.76–1.16	mm

* *t*-test analysis of longevity: males survived longer than females when fed honey, and females had access to hosts (t_78_ = 1.75, *p* = 0.042). ** *t* test analysis for sex ratio % ♀ offspring was significantly higher than % ♂ offspring (t_46_ = −t 6.166, *p* < 0.0001). n = number of replicates.

**Table 3:** Results of no-choice host specificity assessments for the parasitoid *Aroplectrus dimerus* using 25 non-target Lepidoptera species and *Oxyplax pallivitta* as the control.

Species Name	Parasitoid Eggs Deposited on Larvae (Mean ± SEM)	No. Larvae Parasitized	No. Parasitoids Emerging	No. and % Moths of Test Species Emerging
*Podomachla apicalis*	0 b	0	0	20 (100%)
*O. pallivitta* (control)	3.0 ± 0.9 a	10 (50%)	49 (42 ♀, 7 ♂)	-
*Secusio extensa*	0 b	0	0	15 (75%)
*O. pallivitta* (control)	5.1 ± 1.1 a	13 (65%)	94 (61 ♀, 33 ♂)	-
*Choreutis* sp.	0 b	0	0	15 (75%)
*O. pallivitta* (control)	4.1 ± 0.9 a	12 (60%)	73 (52 ♀, 21 ♂)	-
*Diaphania nitidalis*	0 b	0	0	20 (100%)
*O. pallivitta* (control)	3.4 ± 0.7 a	14 (70%)	67 (52 ♀, 23 ♂)	-
*Omiodes blackburni*	0 b	0	0	14 (70%)
*O. pallivitta* (control)	4.5 ± 1.2 a	11 (55%)	87 (62 ♀, 25 ♂)	-
*Udea stellata*	0 b	0	0	20 (100%)
*O. pallivitta* (control)	5.1 ± 1.1 a	12 (60%)	38 (26 ♀, 12 ♂)	-
*Ethmia nigroapicella*	0 b	0	0	15 (75%)
*O. pallivitta* (control)	3.5 ± 0.8 a	13 (65%)	16 (10 ♀, 6 ♂)	-
*Anacamptodes fragilaria*	0 b	0	0	0 (0%) ^1^
*O. pallivitta* (control)	4.9 ± 0.9 a	17 (85%)	36 (18 ♀, 18 ♂)	-
*Macaria abydata*	0 b	0	0	4 (20%)
*O. pallivitta* (control)	3.6 ± 0.9 a	12 (60%)	27 (20 ♀, 7 ♂)	-
*Lampides boeticus*	0 b	0	0	20 (100%)
*O. pallivitta* (control)	5.5 ± 1.0 a	16 (80%)	45 (28 ♀, 17 ♂)	-
*Achaea janata*	0 b	0	0	19 (95%)
*O. pallivitta* (control)	4.7 ± 1.0 a	14 (70%)	88 (54 ♀, 34 ♂)	-
*Agrotis* sp.	0 b	0	0	1 (5%) ^1^
*O. pallivitta* (control)	3.7 ± 1.2 a	11 (55%)	49 (22 ♀, 29 ♂)	-
*Anomis flava*	0 b	0	0	20 (100%)
*O. pallivitta* (control)	4.8 ± 0.9 a	13 (65%)	91 (61 ♀, 30 ♂)	-
*Heliothis virescens*	0 b	0	0	13 (65%)
*O. pallivitta* (control)	5.1 ± 1.4 a	9 (45%)	60 (38 ♀, 22 ♂)	-
*Pandesma anysa*	0 b	0	0	18 (90%)
*O. pallivitta* (control)	5.3 ± 1.1 a	14 (70%)	101 (70 ♀, 31 ♂)	-
*Spodoptera mauritia*	0 b	0	0	19 (95%)
*O. pallivitta* (control)	4.2 ± 0.9 a	14 (70%)	80 (67 ♀, 13 ♂)	-
*Agraulis vanillae*	0 b	0	0	1 (5%) ^1^
*O. pallivitta* (control)	4.5 ± 0.9	14 (70%)	76 (46 ♀, 30 ♂)	-
*Vanessa cardui*	0 b	0	0	19 (95%)
*O. pallivitta* (control)	4.4 ± 1.0 a	12 (60%)	68 (45 ♀, 23 ♂)	-
*Pieris rapae*	0 b	0	0	19 (95%)
*O. pallivitta* (control)	4.4 ± 1.0 a	13 (65%)	68 (39 ♀, 29 ♂)	-
*Plutella xylostella*	0 b	0	0	20 (100%)
*O. pallivitta* (control)	3.5 ± 1.3 a	8 (40%)	44 (28 ♀, 16 ♂)	-
*Hellula undalis*	0 b	0	0	10 (50%)
*O. pallivitta* (control)	4.8 ± 1.0 a	12 (60%)	56 (33 ♀, 23 ♂)	-
*Daphnis nerii*	0 b	0	0	18 (90%)
*O. pallivitta* (control)	4.6 ± 0.9 a	15 (75%)	67 (6 ♀, 61 ♂)	-
*Croesia zimmermani*	0 b	0	0	19 (95%)
*O. pallivitta* (control)	2.6 ± 0.8 a	8 (40%)	13 (9 ♀, 4 ♂)	-
*Cryptophlebia ombrodelta*	0 b	0	0	20 (100%)
*O. pallivitta* (control)	3.0 ± 0.9 a	11 (55%)	33 (24 ♀, 9 ♂)	-
*Episimus utilis*	0 b	0	0	20 (100%)
*O. pallivitta* (control)	3.9 ± 0.5 a	17 (85%)	42 (23 ♀, 19 ♂)	-

^1^ Disease or undetermined cause prevented larvae from completing development. Means followed by different letters are significantly different (ANOVA, *p* < 0.05). F_1,39_ = 11.9, *p* = 0.0014.

**Table 4:** Colonization of *Aroplectrus dimerus* for biocontrol of *Oxyplax pallivitta* on the Hawaiian Islands (2010–2023).

Island	Locality	Release Period	Major Infestation Release Sites Geographic Coordinates and Elevation	Release Sets and Range Wasps/Lot	Total Wasp Released
Oahu Island	Waimanalo, Winward Oahu 2010	17 May 2010– 18 November 2010	Ahiki, (21°20′08.42″ N, 157°42′58.88″ W, 27 m AMSL)	12 (50–441)	1494
“	Waimanalo 2010	9 October 2010– 3 December 2010	Leilani Nursery, (21°20′32.52″ N, 157°43′26.16″ W, 21 m AMSL) C and L Nursery, (21°19′38.78″ N, 157°42′57.10″ W, 89 m AMSL)	5 (50–110)	310
“	Waimanalo 2011	13 January 2011– 8 June 2011	Ahiki, (21°20′08.42″ N, 157°42′58.88″ W, 27 m AMSL)	5 (50–100)	400
“	Central Oahu 2010	8 June 2010– 1 November 2010	Kipapa Gulch, 21°27′32.80″ N, 158°00′57.32″ W, 215 m AMSL) Uka Elem. Sch, Mililani (21°26′14.33″ N, 158°00′55.49″ W, 179 m AMSL) Poloahilani St, Mililani (21°26′51.10″ N, 158°00′12.34″ W, 212 m AMSL)	12 (20–386)	849
“	Central Oahu 2011	2 February 2011– 14 June 2011	Noholoa Park, Mililani (21°26′29.16″ N, 158V 00′29.55″ W, 184 m AMSL) Takenaka’s Nursery, Wahiawa (21°25′44.31″ N, 158°00′55.56″ W, 151 m AMSL)	5 (50–200)	610
“	Windward Oahu 2021	3 October 2021– 16 December 2021	Olomana (21°21′55.00″ N, 157°44′22.61″ W, 56 m AMSL) Lanikai (21°23′30.78″ N, 157°42′56.97″ W, 5 m AMSL) Enchanted lake, (21°23′05.17″ N, 157°44′04.84″ W, 5 m AMSL)	9 (40–70)	526
“	Winward and North Oahu 2022	15 February 2022– 20 December 2022	Haleiwa (21°35′33.15″ N, 158°06′12.88″ W, 1m AMSL) Pauahilani st. kaillua (21°23′29.96″ N, 157°43′27.53″ W, 21 m AMSL)	22 (50–100)	1465
“	Winward and Central Oahu 2023	6 February 2023– 23 August 2023	Maunawili (21°22′50.57″ N, 157°45′22.49″ W, 37 m AMSL) Kaululena St. Mililani (21°27′17.52″ N, 158°00′13.31″ W, 222 m AMSL)	8 (40–220)	880
Hawaii Island	North Kona 2010	5 August 2010– 30 October 2010	3-Ring ranch, Kailua, Kona (19°38′37.21′N, 155°57′55.67″ W, 252 m AMSL) Loloa Way (19°43′29.75″ N, 155°59′27.00″ W, 325 m AMSL) Hawaiian Sunshine (21°20′26.44″ N, 157°43′0324″ W, 13 m AMSL)	7(40–100)	420
“	North Hilo and Puna 2010	16 June 2010– 12 November 2010	Umauma, (19°54′18.50″ N, 155°08′28.8″ W, 115 m AMSL) Kurtistown, (19°35′34.87″ N, 155°03′27.95″ W, 200 m AMSL) Stainback (UH Exptl. Sta.) (19°39′11.48″ N, 155°02′58.20′W, 77 m AMSL)	7 (50–250)	800
“	Kona, North Kohala, North, South Hilo 2011	16 January 2011– 21 March 2011	Puna Orchids, Kapoho (19°29′50.97″ N, 154 °57′03.03″ W, 190 m AMSL) Kohala (20°14′15.25″ N, 155°49′07.74″ W, 158 m AMSL) Onomea (19°48′31.67″ N, 155°05′45.24″ W, 87 m AMSL) Akaka Falls (19°51′14.25″ N, 155°09′07.47″ W, 366 m AMSL) Panaewa, Umauma (19°39′34.28″ N, 155°02′50.93″ W, 57 m AMSL)	31 (50–100)	2725
“	Hilo, Puna, districts 2011	27 January 2011– 8 February 2011	Pahua (19″ 27′41.80″ N, 154″ 56′25.71 W, 294 m) Panaewa, (19°39′34.28″ N, 155°02′50.93″ W, 57 m AMSL) Kurtistown, (19°35′34.87″ N, 155°03′27.95″ W, 200 m AMSL)	4 (50)	200
Maui Island	North shore and East Maui 2010	3 August 2010– 23 November 2010	Haiku (20°55′02.87″ N, 156°19′32.93″ W, 144 m AMSL) Hana, Maliko Gulch (20°55′54.48″ N, 156°20′19.66″ W, 11 m AMSL)	9 (50–200)	910
“	North Shore 2011	25 February 2011– 26 August 2011	Twin Falls off, Hana. (20°54′43.80″ N, 156°14′34.31″ W, 148 m AMSL)	6 (50–150)	640
Kauai Island	East and North districts 2010	19 October 2010	Kuamoo Rd, Kapaa (22°03′27.94″ N, 159°22′52.16″ W, 98 m AMSL) Kiluea, Kauai Orchids (22°11′51.05″ N, 159°22′34.09″ W, 109 m AMSL)	5 (15–100)	200
“	East district 2011	5 May 2011– 30 August 2011	Kapaa, Transfer Station (22°05′00.14″ N, 159°19′26.43″ W, 21 m AMSL)	15 (25–100)	950
Total releases and mean/locality				162 (release sites)	13,379 836.2 ± 158.8

**Table 5:** *Oxyplax pallivitta* infestation and parasitism rates on the island of Oahu, 2019–2023. Hyperparasitoid identified as *Pediobius imbreus* (Hymenotera: Eulophidae).

Date	Locality	No. of Samples	Total *Oxyplax* Larvae Collected	*Oxyplax* Larvae/Sample Mean ± SEM	Total Larvae Parasitized by *Aroplectrus*	% Parasitism by *Aroplectrus*	Total Larvae with Hyper Parasitoid	% Parasitism by *Pediobius*
10 September 2019	Enchanted lake, Kailua	1	52	52	0	0	-	-
24 September 2009– 23 December 2019	Lanikai	12	812	67.7 ± 8.9	52	6.4	5	9.6
23 October 2019– 10 December 2019	Waimanalo	3	125	41.7 ± 7.9	10	8.0	1	10.0
27 November 2019	Kipapa Gulch	1	1	1	0	-	-	-
27 January 2020– 18 October 2020	Lanikai	4	77	19.3 ± 4.1	16	20.8	4	25.0
28 July 2020	Kailua	1	20	20	0	-	-	-
16 October 2020– 16 December 2020	Maunawili Loop, Kailua	3	261	87.0 ± 27.2	2	0.8	2	100
23 January 2020	Waimanalo	3	54	18.0 ± 7.2	5	9.2	5	100
4 January 2021– 26 July 2021	Haleiwa	4	47	11.7 ± 6.7	3	6.4	1	33.3
10 March 2021	Waimanalo	7	58	8.3 ± 3.8	38	65.5	5	13.1
7 April 2021– 11 August 2021	Maunawili, Kailua	6	51	8.5 ± 3.0	0	0	0	-
7 April 2021– 20 October 2021	Lanikai	11	314	28.5 ± 5.9	23	7.3	0	0
5 August 2021– 11 November 2021	Olomana	6	466	77.7 ± 17.9	36	7.7	0	0
14 June 2021– 16 December 2021	Enchanted Lake, Kailua	6	417	69.5 ± 25.3	26	6.2	0	0
19 January 2022– 15 October, 2022	Enchanted lake, Kailua	6	41	6.8 ± 1.8	18	43.9	2	11.1
23 February 2022– 6 December 2022	Maunawili	2	24	12.0 ± 8.0	0	0	0	-
11 January 2022 – 20 December 2022	Lanikai	8	239	29.9 ± 7.0	26	10.9	1	3.8
19 January 2022– 26 September 2022	Olomana	8	262	32.7 ± 7.7	121	46.2	65	53.7
9 March 2022– 12 July 2022	Waimanalo	2	2	2	2	100	0	0
10 November 2022	kaillua	1	13	13	0			
10 January 2023– 23 October 2023	Lanikai	8	248	31.0 ± 9.1	12	4.8	1	8.3
18 January 2023– 31 October 2023	Olomana	5	125	25.0 ± 7.0	70	56.0	27	38.6
18 January 2023– 28 July 2023	Enchanted lake, Kailua	2	5	2.5 ± 0.5	0	0	0	
28 April 2023– 23 August 2022	Mililani	4	59	14.7 ± 6.4	5	8.5	3	60.0
6 February 2023– 21 August 2023	Waimanalo	2	50	25.0 ± 13.0	4	8.0	1	25.0
Mean ± SEM /site				28.22 ± 4.9	18.76 ± 5.6	18.93 ± 5.6	5.86 ± 3.2	27.30 ± 7.6
All samples		117 samples (25 sites)	3923 larvae		469 larvae		123 larvae	

## Data Availability

The data presented in this study are available on request from the corresponding author.

## Supplementary Materials

The following supporting information can be downloaded at: https://www.mdpi.com/article/10.3390/life14040509/s1, Figure S1: Distribution pattern of Aroplectrus eggs count per host larva.
