# Supplementary figures and images for: Aroplectrus dimerus (Hymenoptera: Eulophidae), Ectoparasitoid of the Nettle Caterpillar, Oxyplax pallivitta (Lepidoptera: Limacodidae): Evaluation in the Hawaiian Islands
Source: Life (Basel). 2024 Apr 15;14(4):509. doi: 10.3390/life14040509 (PMC11051351; doi:10.3390/life14040509)

# Aroplectrus eggs deposited per Darna larva

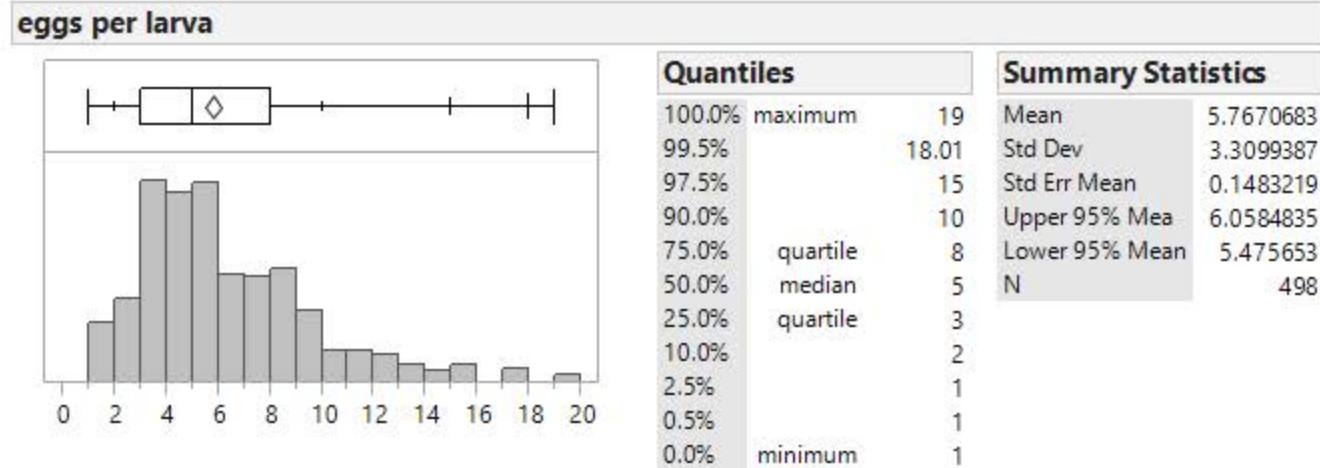

Supplement: Supplementary file 1 [file life-14-00509-s001.zip › life-2937578-supplementary.pdf]
